# A risk model for detecting clinically significant prostate cancer based on bi-parametric magnetic resonance imaging in a Japanese cohort

**DOI:** 10.1038/s41598-021-98195-2

**Published:** 2021-09-22

**Authors:** Kazushige Sakaguchi, Michikata Hayashida, Naoto Tanaka, Suguru Oka, Shinji Urakami

**Affiliations:** Department of Urology, Toranomon Hospital, 2-2-2- Toranomon, Minato-ku, Tokyo, 105-8470 Japan

**Keywords:** Cancer screening, Urological cancer

## Abstract

Selective identification of men with clinically significant prostate cancer (sPC) is a pivotal issue. Development of a risk model for detecting sPC based on the prostate imaging reporting and data system (PI-RADS) for bi-parametric magnetic resonance imaging (bpMRI) and clinical parameters in a Japanese cohort is expected to prove beneficial. We retrospectively analyzed clinical parameters and bpMRI findings from 773 biopsy-naïve patients between January 2011 and December 2016. A risk model was established using multivariate logistic regression analysis and presented on a nomogram. Discrimination of the risk model was compared using the area under the receiver operating characteristic curve. Statistical differences between the predictive model and clinical parameters were analyzed using DeLong test. sPC was detected in 343 men (44.3%). Multivariate logistic regression analysis to predict sPC revealed age (*P* = 0.002), log prostate-specific antigen (*P* < 0.001), prostate volume (*P* < 0.001) and PI-RADS scores (*P* < 0.001) as significant contributors to the model. Area under the curve was higher for the risk model (0.862), than for age (0.646), log prostate-specific antigen (0.652), prostate volume (0.697) or imaging score (0.822). DeLong test results also showed that the novel risk model performed significantly better than those parameters (*P* < 0.05). This novel risk model performed significantly better compared with PI-RADS scores and other parameters alone, and is thus expected to prove beneficial in making decisions regarding biopsy on suspicion of sPC.

## Introduction

Prostate cancer is the most commonly diagnosed cancer in Japan. The incidence of prostate cancer is rapidly increasing, with over 90,000 men newly diagnosed in 2017. Over 12,000 men died of prostate cancer in 2018, representing the 6th-most frequent cause of cancer-related death among men in Japan^1^. Population-based prostate-specific antigen (PSA) screening tests can facilitate early detection of prostate cancer and thus lead to declines in prostate cancer related-mortality^2^. However, these tests simultaneously lack specificity, resulting in increased numbers of unnecessary prostate biopsies, which in turn are associated with risks of rectal bleeding and sepsis. The risk of over-treatment leading to adverse impacts on quality of life without improving survival is a concern. Randomized controlled clinical studies that evaluated the efficacy of prostate cancer screening have highlighted the need to reduce the over-diagnosis of clinically insignificant prostate cancer. A new diagnostic pathway is thus needed to selectively identify men with clinically significant prostate cancer (sPC), while reducing the number of unnecessary biopsies and over-detection and over-treatment of clinically insignificant prostate cancer^2,3^.

The use of multi-parametric magnetic resonance imaging (mpMRI) of the prostate, incorporating anatomical and functional imaging (T2-weighted imaging, diffusion-weighted imaging (DWI) and dynamic contrast enhancement (DCE)) appears beneficial for detecting sPC. However, mpMRI has been criticized for widely variable diagnostic performance across different institutions. In 2012, Prostate Imaging Reporting and Data System (PI-RADS) was introduced to facilitate standardized interpretation of mpMRI findings^4^. In PI-RADS, a score for suspecting the presence of sPC is assigned on a 5-point scale for the mpMRI sequence. PI-RADS has shown high diagnostic accuracy for detecting sPC by means of targeted biopsies^5,6^.

The use of clinical data with mpMRI findings has become significantly important for urologists to better stratify individuals who may warrant prostate biopsy. Multivariable prediction models are superior to conventional decision-making based solely on PSA testing or digital rectal examination (DRE) in predicting the outcomes of prostate biopsies. Previous multivariable prediction models for detecting sPC were based on clinical parameters including various combinations of age, PSA, prostate volume (PV), DRE findings and others. MRI findings were also utilized as a parameter of prediction models, but without a standardized reporting system^7,8^. The utility of an individualized risk calculator and a multivariable nomogram (a nomogram is a graphical calculating device, specifically the approximate probability of sPC derived by mathematical logistic function in this study) including data from mpMRI using PI-RADS score for detecting sPC have been reported^9–11^. Furthermore, the use of bi-parametric MRI (bpMRI) of the prostate incorporating anatomical and functional imaging (T2-weighted imaging and DWI not containing DCE) has been shown to maintain high diagnostic accuracy^12,13^. Predictive models based on bpMRI findings and clinical parameters for risk assessment and selection of sPC have also recently been reported^14,15^.

However, epidemiologically, the characteristics of prostate cancer exhibit regional and ethnic differences^16^. While risk calculators and nomograms should ideally be structured from the same cohorts with good validation, no reports have described a risk calculator and nomogram using PI-RADS scores combined with other clinical parameters from a Japanese-only cohort^6^. The aim of the present study was to develop the first risk model and nomogram using PI-RADS score among Japanese men for detecting sPC and reducing the over-detection and over-treatment of clinically insignificant prostate cancer.

## Methods

### Study population

A total of 773 biopsy-naïve patients from a single institution (Toranomon hospital, Tokyo, Japan) between January 2011 and December 2016 and suspected to have localized prostate cancer based on abnormal PSA levels were analyzed retrospectively. Indications for biopsy were high PSA level (≥ 4.0 ng/ml), abnormal DRE or lesions suggestive of prostate cancer on bpMRI. Exclusion criteria were previous prostate surgery, previous diagnosis of prostate cancer or administration of 5-alpha-reductase inhibitors or anti-androgens, as agents that affect PSA values. Full data on PI-RADS scores of bpMRI, biopsy outcome, PSA, age and PV were available for all patients. Data from bpMRI performed before the introduction of PI-RADS were reinterpreted and new PI-RADS scores were assigned. Those samples were used for development and internal validation of the risk model. The study was approved by Toranomon Hospital Ethics Committee (approval no. 1573). All methods were conducted in accordance with the relevant local guidelines and regulations. All patients provided informed consent or were informed that the hospital web-page included an opt-out option, as approved by the Toranomon Hospital Ethics Committee.

### Imaging

All bpMRI was performed using a 1.5- or 3.0-T system (Magnetom; Siemens, Erlangen, Germany) with a multichannel body surface coil. The bpMRI protocol included axial, coronal and sagittal turbo spin echo T2-weighted sequences and axial DWI with apparent diffusion coefficient (ADC) calculation (Supplementary Table S1). ADC maps were rebuilt in each pixel of each slice using the mono-exponential model. A 1.5-T system was generally used for the first bpMRI and a 3.0-T system was used for the second and subsequent bpMRI. All image analyses were performed according to PI-RADS version 2.0 on a scale from 1 to 5, with higher numbers indicating a greater likelihood of sPC^17^. Analyses were performed by or under the supervision of a few expert uroradiologists. Overall, PI-RADS scores for each lesion were determined separately for the peripheral zone and transitional zone, entailing assignment of separate scores for each of the T2-weighted and DWI sequences. PV was calculated on T2-weighted imaging, calculated as 0.52 × length × width × height.

### Biopsy protocol

All patients underwent systematic transperineal and transrectal biopsy (mapping 8–14 cores) of the whole gland in the lithotomy position under local anesthesia, carried out by one of several expert urologists^18^. The number of needle cores was decided by prostate size and risk of infection associated with rectal biopsy for each patients. If one or more lesions suggestive of prostate cancer were detected on bpMRI (suspicious lesions were consistent with PI-RADS score ≥ 3 retrospectively), transperineal cognitive targeted biopsies were added for each lesion (2–4 cores of each lesion; median, 2 per lesion). Transrectal ultrasound echography (ARIETTA; Hitachi Aloka Medical, Wallingford, CT, USA) was used to guide biopsies without MRI fusion software.

### Histopathology

Histopathological analyses from biopsies were performed by or under the supervision of a few expert uropathologists specializing in prostate assessment according to International Society of Urological Pathology standards. For all cores, the length of the cancer in millimeters and both primary and secondary Gleason grades were assigned separately. The study defined sPC as grade group ≥ 3 (Gleason score ≥ 4 + 3) or a maximum cancer core length ≥ 6 mm in any location^5^.

### Statistical analysis

Patient demographics, MRI and biopsy results (age, PSA, PV, PI-RADS score 1–5 and presence or absence of sPC) were analyzed descriptively. First, we divided all patients into two groups by pathological outcome: a sPC group; and an others group. The others group included patients with clinically insignificant prostate cancer or no cancerous tissue. Clinical parameters were compared between groups using the Wilcoxon test and Pearson test. Consequently, we performed multivariate logistic regression analysis to predict the presence of sPC on biopsy. We calculated odds ratios and used multivariate logistic regression-based coefficients to develop multivariable nomograms for predicting the probability of sPC (a nomogram is a graphical calculating device, specifically the approximate probability of sPC derived by mathematical logistic function in this study). To avoid linearity assumptions, PSA was transformed into the logarithmic PSA.

The discrimination of risk models for sPC with or without MRI scoring was compared using the area under the curve (AUC) of receiver operating characteristic (ROC) curves. The significance of differences between predictive models was analyzed using DeLong test.

The extent of over- or underestimation of the predicted rate relative to the observed rate of sPC was explored graphically using calibration plots, which were internally validated using 1000 bootstrap resamples. The intercept indicates whether predictions are systematically too low or too high, and thus should ideally be zero. The calibration slope reflects the average effects of predictors in the model and is estimated in a logistic regression model with the logit of model predictions as the only predictor. For a perfect model, the slope equals 1^19^.

Last, we assessed the performance of the risk model for its clinical usefulness using decision curve analysis (DCA) based on bootstrapped validation repeated 1000 times. These analyses estimate a ‘net benefit’ for prediction models by totaling the benefits (true-positive biopsies) and subtracting the harms (false-positives biopsies)^20^. Harms are weighted by the relative harm of a missed sPC compared to unnecessary biopsy. The weighted rate is derived from the threshold probability of sPC at which a patient would opt for biopsy. This threshold can thus vary from patient to patient in clinical settings. The reduction in number of biopsies using different probabilities was further assessed and related to the number and percentage of sPC detected. Interpretation of the decision curve was based on the model with the highest net benefit at a particular threshold probability representing the most useful model for risk and benefit. To quantify the potential reduction of unnecessary biopsies and potential over-diagnosis, we calculated true-positive rate (TPR), false-positive rate (FPR), positive predictive value (PPV) and negative predictive value (NPV) at exemplary probability thresholds.

All tests performed were two-sided and values of *P* < 0.05 were considered to indicative of statistical significance. Statistical analyses were performed using R version 4.0.2 (R Foundation for Statistical Computing, Vienna, Austria). ROC analysis and DCA were performed utilizing the pROC package and rmda package, respectively. Reporting followed the Standards of Reporting of Diagnostic Accuracy (Supplementary Tables S2, S3).

## Results

In total, sPC was detected in 343 men (44.3%). Demographic characteristic, and MRI and biopsy data from both groups are given in Table 1. Men in the sPC group were older (median age, 69 years vs 65 years, *P* < 0.001), had higher PSA (median PSA, 9.01 ng/ml vs 6.72 ng/ml, *P* < 0.001), and had lower PV (median PV, 29.6 ml vs 39.85 ml, *P* < 0.001). The proportion of borderline and malignant lesions on bpMRI (PI-RADS score 3, 4 or 5) was significantly higher in the sPC group (93.59% vs 65.46%, *P* < 0.001).

**Table 1:** Clinical parameters and MRI PI-RADS score both 2 groups.

Parameter	sPC group n = 343	Others group n = 430	*P* value
Median age, year (IQR)	69 (64–75)	65 (60–70)	< 0.001
Median PSA, ng/ml (IQR)	9.01 (6.32–13.40)	6.72 (5.26–9.44)	< 0.001
Median PV, ml (IQR)	29.60 (23.80–37.25)	39.85 (29.75–51.77)	< 0.001
Number of cores, median (IQR)	8 (8–14)	8 (8–9)	0.021
PI-RADS score	Score 1 n = 3 (0.87%)	Score 1 n = 8 (1.42%)	< 0.001
	Score 2 n = 19 (5.54%)	Score 2 n = 237 (33.12%)	
	Score 3 n = 94 (27.41%)	Score 3 n = 111 (26.52%)	
	Score 4 n = 98 (28.57%)	Score 4 n = 44 (18.37%)	
	Score 5 n = 129 (37.61%)	Score 5 n = 30 (20.57%)	

*IQR* interquartile range, *PI-RADS* prostate imaging reporting and data system, *SA* prostate-specific antigen, *PV* prostate volume, *sPC* clinically significant prostate cancer.

**Figure 1:**
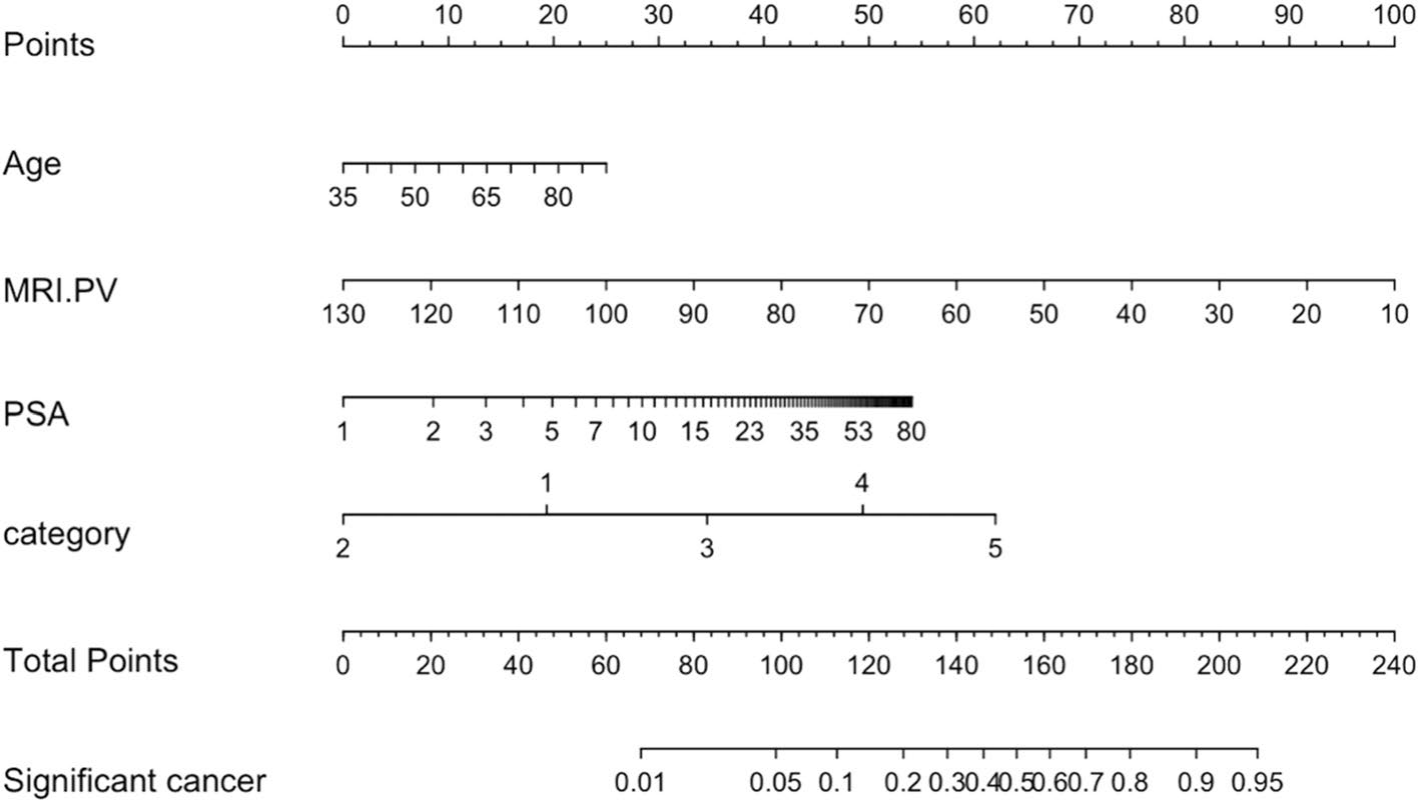
Risk model to predict sPC including age, PSA, PV and bpMRI PI-RADS score .

Multivariate logistic regression analysis to predict sPC identified age (*P* = 0.002), logPSA (*P* < 0.001), PV (*P* < 0.001) and PI-RADS score (*P* < 0.001) as significant contributors to the model (Table 2). Multicollinearity was tested between all variables by the individual variance inflation factor and no multicollinearity was found. The nomogram of the risk model and the regression equation are shown in Fig. 1.

**Table 2:** Multivariate logistic regression model analysis for the prediction of sPC.

Parameter	Odds ratio	95% CI	*P* value
Age (per 5 year)	1.214	1.074–1.374	0.002
Log PSA (ng/ml)	2.101	1.441–3.120	< 0.001
PV ( per 10 ml)	0.68	0.591–0.777	< 0.001
**PI-RADS score **			< 0.001
PI-RADS score 2: score 1	0.292	0.073–1.477	0.098
PI-RADS score 3: score 1	2.005	0.532–9.725	0.332
PI-RADS score 4: score 1	4.694	1.220–23.11	0.033
PI-RADS score 5: score 1	6.178	1.552–31.16	0.014

*CI* confidence interval, *PI-RADS* prostate imaging reporting and data system, *PSA* prostate-specific antigen, *PV* prostate volume, *sPC* clinically significant prostate cancer.

**Figure 2:**
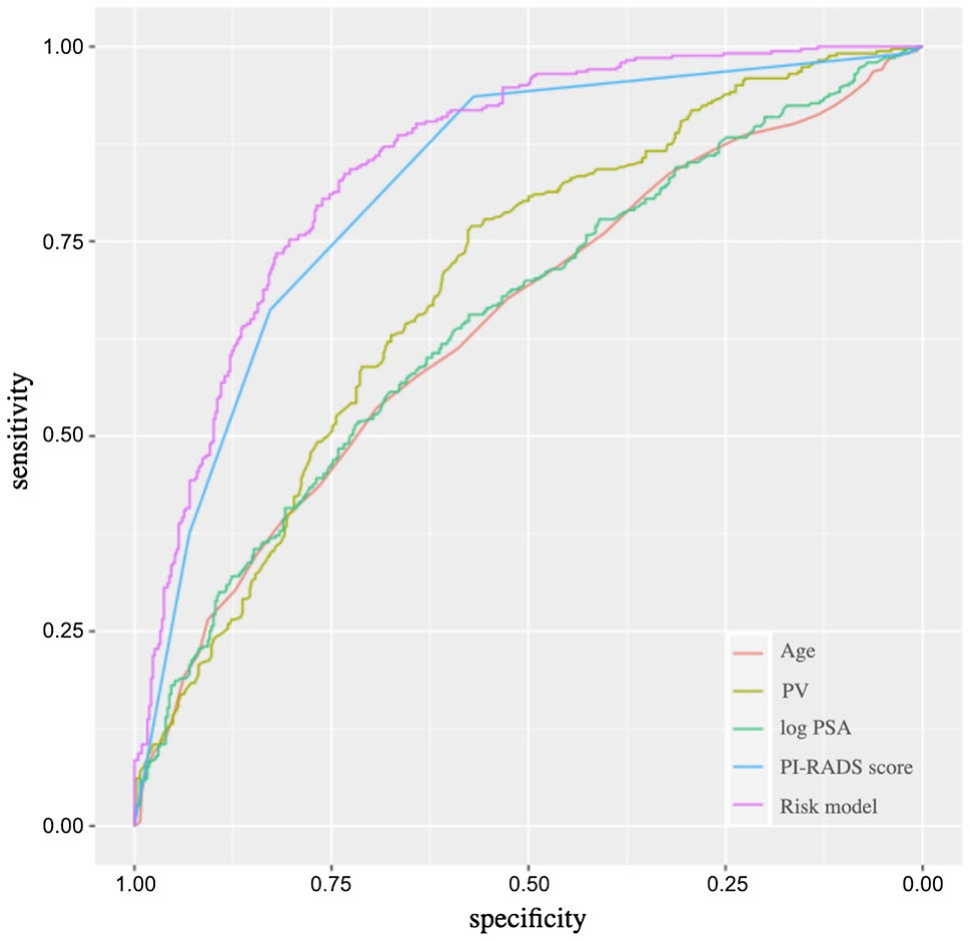
ROC curve analysis for the performance of age (red line), PV (yellow line), PSA (green line), bpMRI PI-RADS (blue line) and risk model (purple line) to predict sPC.

**Figure 3:**
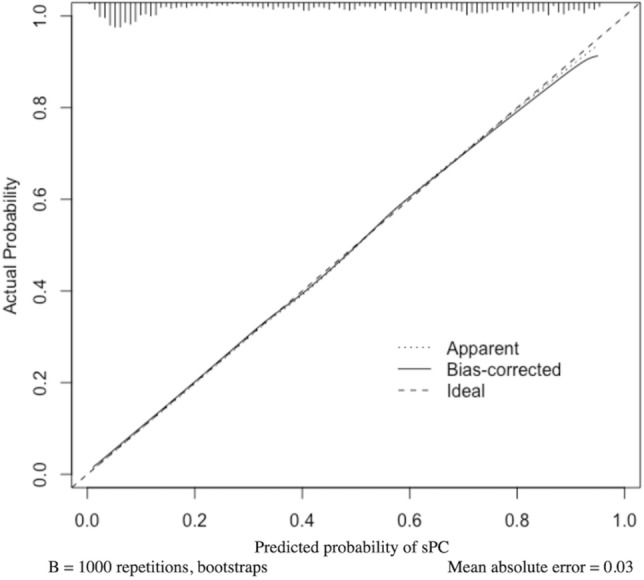
Calibration plots for the risk models to predict sPC.

The novel risk model was internally validated by bootstrapping. Discrimination of the risk model was compared using parameters included in ROC analyses (Fig. 2, Table 3). AUC was higher for the risk model (0.862), than for age (0.646), PV (0.697), logPSA (0.652) or PI-RADS score (0.822). DeLong test results also showed that the novel risk model performed significantly better compared with those parameters including PI-RADS score alone (Table 3). Table4 shows TPR, FPR, PPV and NPV at exemplary probability thresholds of this risk model and the optimal PI-RADS score cutoff. At a probability threshold of 10%, the net reduction in biopsies taken based on the risk model was 43.0%, while the rate of missing sPC was 2.3%. Bootstrapped calibration plots of the risk model demonstrated no untoward deviations of predicted risk from observed risk of sPC over the entire range (Fig. 3).

**Table 3:** AUC of ROC curve analysis for the performance of age, PV, PSA, PI-RADS and risk model to sPC, and DeLong test for model and factors comparison.

	AUC	95% CI
**Parameter**		
Age	0.646	0.607–0.685
PV	0.697	0.661–0.734
log PSA	0.652	0.613–0.691
PI-RADS score	0.822	0.793–0.851
Risk model	0.862	0.837–0.888

	*P* value	
**Comparison of models and factors**		
Risk model versus Age	< 0.001	
Risk model versus PV	< 0.001	
Risk model versus log PSA	< 0.001	
Risk model versus PI-RADS score	0.039	

*AUC* area under the curve, *CI* confidence interval, *PI-RADS* prostate imaging reporting and data system, *PSA* prostate-specific antigen, *PV* prostate volume, *ROC* reciever operating characteristic, *sPC* clinically significant prostate cancer.

**Table 4:** Prediction errors for diagnosis of sPC as 5%, 10%, 20%, 50% and best cut-offs for risk model and PI-RADS score .

Risk model	TPR	FPR	PPV	NPV
**Parameter**				
5% probability of sPC cut-off	0.994	0.811	0.496	0.976
10% probability of sPC cut-off	0.977	0.57	0.579	0.958
20% probability of sPC cut-off	0.936	0.43	0.636	0.917
50% probability of sPC cut-off	0.787	0.217	0.744	0.821
Best cut-off of risk model: 41% probability of sPC	0.863	0.271	0.718	0.869
Best cut-off of PI-RADS: score between 2 and 3	0.936	0.43	0.634	0.918

*FPR* false positive rate, *NPV* negative predictive value, *PI-RADS* prostate imaging reporting and data system, *PPV* positive predictive value, *sPC* clinically significant prostate cancer, *TPR* true positive rate.

**Figure 4:**
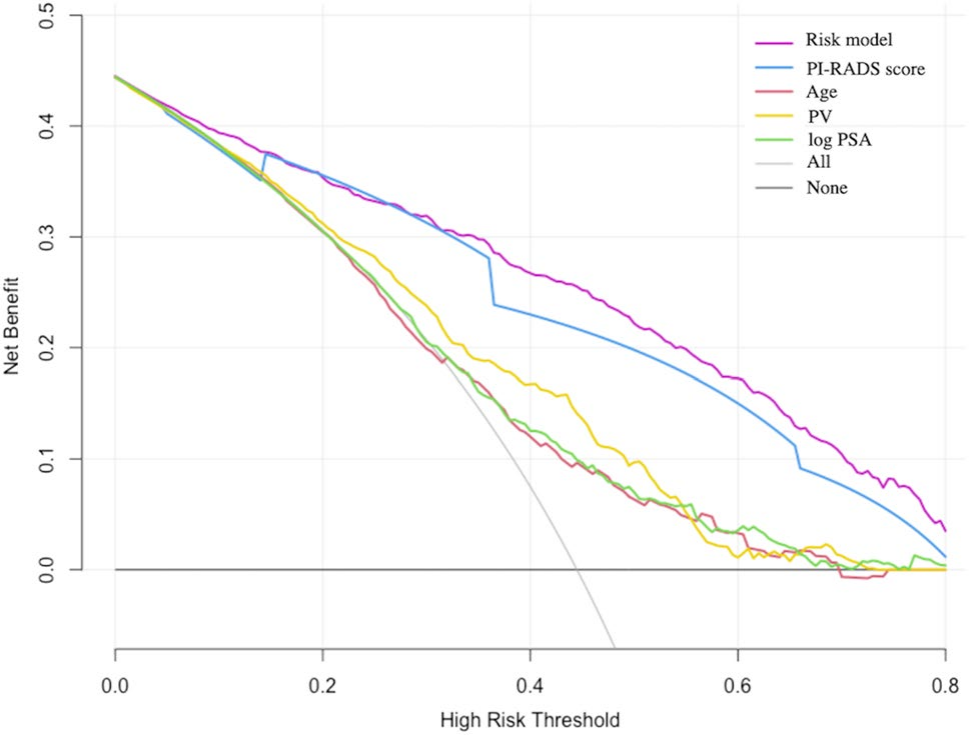
Net DCA demonstrating the benefit for predicting sPC on biopsy.

In bootstrapped DCA, the risk model showed a higher net benefit in terms of accurately detecting patients with sPC, compared with PI-RADS score and other parameters alone (Fig. 4). The risk model showed a benefit for sPC threshold probabilities larger than 10%.

## Discussion

While mpMRI can detect 85–95% of sPC compared with prostatectomy specimens, the sensitivity, NPV and specificity of mpMRI have been reported as 58–96%, 63–98% and 23–87%, respectively^5,21^. Because of the high diagnostic accuracy for sPC detection, upfront mpMRI has been recommended as a triage test to indicate the need for biopsy among biopsy-naïve men in whom sPC was suspected due to high PSA^22–24^. As a result of the high NPV, men with no suspected evidence of sPC on MRI may defer systematic biopsy^25^. Moreover, to improve predictive values, new multivariate risk prediction tools have recently been constructed using the mpMRI suspicion score ^9,10,26^.

The bpMRI procedure involves performing prostate MRI without DCE, and produces beneficial results. The effectiveness of bpMRI detecting sPC in biopsy-naïve patients has been reported. In addition, bpMRI has the advantage of avoiding the adverse events associated with some gadolinium-based contrast agents, as well as shortened examination time and reduced costs^27^. On the other hand, DCE MRI has been reported to improve the sensitivity of MRI for detecting sPC. At the same time, predictive models based on bpMRI findings and clinical parameters for risk assessment and selection of sPC have also been reported recently^14,15,28,29^.

In a Japanese cohort, the efficacy of mpMRI and bpMRI for detecting sPC as a triage test has been reported^30–32^. However, no multivariate risk prediction models for detecting sPC based on PI-RADS scores of mpMRI or bpMRI as ordinal variables among Japanese populations have been described previously.

The characteristics of our novel risk model were as follows. First, in all cases, bpMRI was performed on the pre-biopsy setting, because biopsy artifacts could affect bpMRI findings and this model was constructed to reduce unnecessary biopsies. Second, variables of DRE and PSA density used in other nomograms were not included in this study. Because anterior prostate cancer is less commonly palpable, use of DRE as a variable in the prediction model means that the dataset of the model should ideally be divided into two groups according to whether DRE findings are positive or not, and each model should be constructed independently^33^. The small size of our dataset did not allow division into two groups. The parameters PSA and PV, and not PSA density, were selected because their interpretations are more explainable and understandable^9^.

PI-RADS score contributed significantly to the model, like other parameters from multivariate logistic regression analysis. Interestingly, the odds ratio of PI-RADS score 2 compared to score 1 was 0.292 (*P* = 0.098), while that of PI-RADS score 3 compared to score 1 was 2.005 (*P* = 0.332) (Table 2). PI-RADS score 1 and score 2 indicated a normal prostate gland and benign prostate disease (inflammatory and/or hyperplasia), respectively. In a proportion of cases with PI-RADS score 2, PSA was elevated because of inflammation and hyperplasia. Therefore, among high-PSA cases, PI-RADS score 1 might carry a higher risk of sPC than PI-RADS score 2 in real clinical practice. Moreover, because of the low number of cases with PI-RADS score 1 (only 11 cases, 1.42%), the odds ratio for PI-RADS score 2 to score 1 might not reach statistical significance. This also explained why lower PV cases tended to carry a higher risk of sPC. This was presumably because multicollinearity among parameters could not be completely excluded even if multivariate analysis was performed.

A low PI-RADS score harbors a 5–10% risk of sPC, allowing biopsy to be potentially avoided^21,34^. Multivariate risk prediction tools including mpMRI findings from regions other than Japan have shown a high AUC of 0.82–0.91^35^. ROC analysis revealed that this novel model offered a high AUC (c index = 0.862) approximately equivalent to previous reports, although this novel model has not been externally validated and should not be compared to other risk models constructed from different regional and ethnic cohorts^9^. The risk model enables avoidance of unnecessary biopsies in more patients without increasing the risk of missing a diagnosis of sPC at an arbitrary probability threshold. More specifically, at probability thresholds of 10% and 20% in this model and with a cut-off PI-RADS score between 2 and 3, net reductions in biopsies were 43.0%, 57.0% and 57.0%, while rates of missing sPC were 2.3%, 6.4% and 6.4%, respectively. Using DCA, the present study showed that the risk model using PI-RADS scores improved clinical decisions for the biopsy of patients with suspected sPC, as compared with clinical parameter models or PI-RADS score alone. The risk model provided benefits in the decision to biopsy patients for sPC at probability thresholds exceeding 10%. From a practical perspective, at various probability cutoffs, the combined models demonstrated the best performance among all prediction parameters. Although cost-effectiveness remains an issue due to differences in social insurance situations and the high penetration rate of MRI in other countries, a protocol for biopsy indications for MRI in cases with high PSA value should be considered.

The present findings should be interpreted in the context of some limitations. First, this study represented a retrospective analysis, thus elevating the risk of selection biases. Second, inter-reader agreement on bpMRI was not evaluated in the present study. Third, low numbers of systemic biopsy cores were collected in our cohort. The number of sPC lesions detected by systemic biopsy was thought to be lower and could have improved model accuracy and internal validation. Fourth, this study targeted the first biopsy cases and the findings thus are not applicable to repeat biopsy cases. Last, no external validation was performed. If the excellent results obtained with bpMRI and other clinical parameters from a single institution as in this study are not reproduced in other hospitals, the broad use of the novel risk model will lead to patient mismanagement in a substantial proportion of cases.

To the best of our knowledge, this represents the first report of a risk calculator and nomogram using PI-RADS version 2 score of bpMRI among Japanese males for detecting sPC in pre-biopsy settings. On the other hand, recent risk models have been reported to detect sPC using quantitative mpMRI, which may also help standardize mpMRI and bpMRI interpretation and image recognition using new statistical tools (machine learning, deep learning and neural network analysis)^36,37^. Risk models using genetic elements and molecular markers rather than image variables are also being reported^38^. Finally, prospective and multi-centric risk models for sPC risk prediction including such new biochemical parameters, financial aspects and novel MRI fusion biopsy data are expected to be established in the future.

## Supplementary Information

Supplementary Table S1.

Supplementary Table S2.

Supplementary Table S3.
